# Hydraulic and Mechanical Impacts of Pore Space Alterations within a Sandstone Quantified by a Flow Velocity-Dependent Precipitation Approach

**DOI:** 10.3390/ma13143100

**Published:** 2020-07-11

**Authors:** Maria Wetzel, Thomas Kempka, Michael Kühn

**Affiliations:** 1 German Research Centre for Geosciences, Fluid Systems Modelling, Telegrafenberg, 14473 Potsdam, Germany; kempka@gfz-potsdam.de (T.K.); michael.kuehn@uni-potsdam.de (M.K.); 2 Institute of Geosciences, University of Potsdam, Karl-Liebknecht-Str. 24/25, 14476 Potsdam, Germany

**Keywords:** Bentheim sandstone, digital rock physics, micro-CT, elastic properties, permeability, precipitation

## Abstract

Geochemical processes change the microstructure of rocks and thereby affect their physical behaviour at the macro scale. A micro-computer tomography (micro-CT) scan of a typical reservoir sandstone is used to numerically examine the impact of three spatial alteration patterns on pore morphology, permeability and elastic moduli by correlating precipitation with the local flow velocity magnitude. The results demonstrate that the location of mineral growth strongly affects the permeability decrease with variations by up to four orders in magnitude. Precipitation in regions of high flow velocities is characterised by a predominant clogging of pore throats and a drastic permeability reduction, which can be roughly described by the power law relation with an exponent of 20. A continuous alteration of the pore structure by uniform mineral growth reduces the permeability comparable to the power law with an exponent of four or the Kozeny–Carman relation. Preferential precipitation in regions of low flow velocities predominantly affects smaller throats and pores with a minor impact on the flow regime, where the permeability decrease is considerably below that calculated by the power law with an exponent of two. Despite their complete distinctive impact on hydraulics, the spatial precipitation patterns only slightly affect the increase in elastic rock properties with differences by up to 6.3% between the investigated scenarios. Hence, an adequate characterisation of the spatial precipitation pattern is crucial to quantify changes in hydraulic rock properties, whereas the present study shows that its impact on elastic rock parameters is limited. The calculated relations between porosity and permeability, as well as elastic moduli can be applied for upscaling micro-scale findings to reservoir-scale models to improve their predictive capabilities, what is of paramount importance for a sustainable utilisation of the geological subsurface.

## 1. Introduction

Geochemical fluid-rock interactions such as precipitation and dissolution of minerals alter the microstructure of rocks, and thereby affect their physical behaviour at the macro scale. The prediction of the resulting changes in effective hydraulic and mechanical rock properties is of paramount importance for numerous natural geochemical systems and commercial applications such as geothermal energy production [1,2], hydrocarbon exploration and exploitation [3,4], nuclear waste disposal [5,6] as well as energy and gas storage [7,8,9]. Within the context of subsurface utilisation, the interaction of hydraulic, mechanical and chemical processes may be triggered or preferably prevented depending on the geological application: Precipitation of minerals reduces the permeability, what could negatively impact the injectivity and productivity of a reservoir [10,11], but on the other hand mineral growth can seal potential leakage pathways, e.g., in the context of CO_2_ storage [12,13]. Regarding changes in mechanical properties, an effective stiffening of rocks due to their cementation [14,15] may be of particular relevance for the integrity of a reservoir or fault systems.

**Figure 1:**
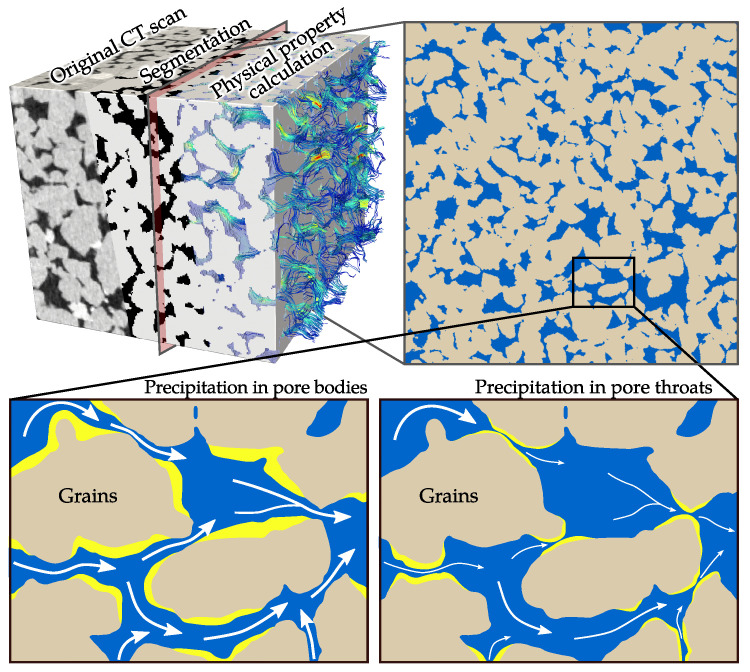
The typical workflow in digital rock physics comprises imaging, segmentation and numerical computation of the rock properties. In view of a virtual laboratory, varying testing conditions or distributions of secondary minerals can be examined for the same sample, e.g., to investigate the effect of spatial precipitation patterns on the evolution of flow properties.

For an adequate prediction of physical rock properties, it is necessary to characterise the initial microstructure including the morphology and connectivity of pores. In general, precipitation reduces the porosity, but the effect on transport properties may be either negligible or significant depending on the specific location within the pore network (Figure 1). The spatial distribution of mineral nucleation and growth is complex and controlled by various factors including chemistry [16,17], transport properties [18,19], mineralogy [20,21], temperature [22,23] and pore morphology [24,25], whereby the impact of each factor depends on the particular process. Moreover, several studies discuss a pore size dependency of precipitation. A preferential mineral deposition is observed for small pores [16,26] or narrow throats [27] as well as larger pores, which is explained by lower supersaturation thresholds due to interfacial energy effects [25].

However, of actual interest within the context of subsurface utilisation is the impact of pore-scale precipitation on hydraulic and mechanical rock properties. Numerous empirical and analytical relationships describe the dependency between porosity and permeability [28,29,30] as well as porosity and elastic moduli [31,32,33]. Many of them assume a simplified microstructure of rocks by packings of spheres, ellipsoids or pipes. By contrast, high-resolution imaging such as micro-computer tomography (micro-CT) provides the opportunity to use a 3D representation of the rock. It depicts the complexity of the pore space regarding the size, shape and roughness of grains as well as the morphology and connectivity of the pores, which are of particular importance for an accurate prediction of physical rock properties [34,35]. The relatively new field of digital rock physics comprises the numerical simulation of flow [36,37,38] and elastic properties [39,40,41], which are directly calculated on the micro-CT image stack (Figure 1). This non-destructive approach allows further to perform multiple experiments considering varying testing conditions or distributions of secondary minerals on the same rock sample, which is another advantage in view of a virtual laboratory.

In the present study, the effect of precipitation on hydraulic and mechanical rock properties is numerically examined for a typical granular reservoir rock, namely the Bentheim sandstone. Changes in pore morphology, permeability as well as bulk and shear moduli are quantified based on a highly resolved micro-scale representation of the rock. The permeability is determined from the flow field by solving the steady-state Stokes equation [42], whereas effective rock stiffness is calculated by a static finite element method [43]. These well-established approaches are used to investigate the impact of pore space alterations by three different spatial patterns of precipitation depending on the local flow velocities: preferentially on grain surfaces exposed to high flow velocities (HFV), low flow velocities (LFV) and uniform mineral growth independent of fluid flow. The calculated relationships between porosity and permeability, as well as porosity and elastic moduli are discussed in the context of widely-used analytical methods. The impact of precipitation on the evolution of effective physical rock properties is especially of interest for a sustainable utilisation of the geological subsurface.

## 2. Material and Methods

### 2.1. Digital Rock Sample and Morphology of the Pore Space

**Figure 2:**
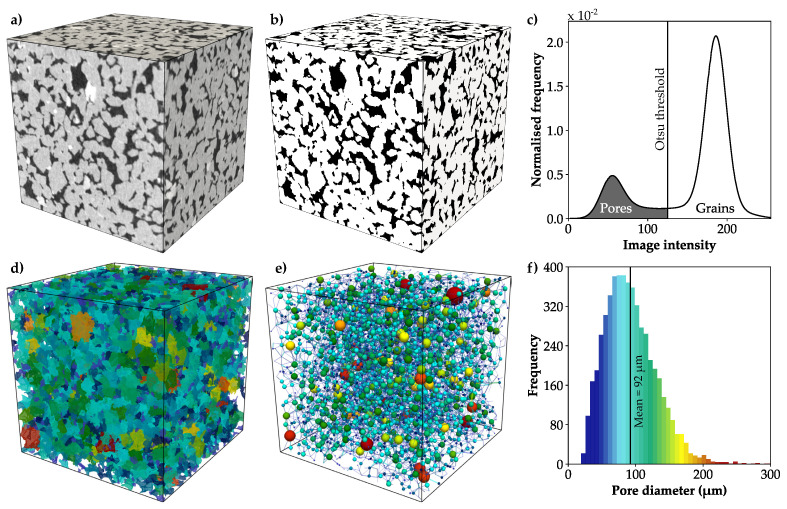
(**a**) Micro-CT scan of the Bentheim sandstone with a size of 500^3^ voxels and a resolution of 4.9 μm. (**b**) The 3D image is binarised by (**c**) an intensity-based separation of the pore space and grains using the Otsu threshold [49]. (**d**) The entire pore space is partitioned into individual pores (coloured by diameter) and (**e**) the pore network is extracted. (**f**) Pore size distribution with a mean diameter of 92 μm.

To obtain a highly resolved three-dimensional pore-scale model of a typical granular reservoir rock, a micro-CT scan of the Bentheim sandstone is chosen. It represents a well-investigated reservoir reference rock and has been examined in numerous studies regarding its mechanical and hydraulic properties [44,45,46], since it is highly porous, homogeneous and isotropic. The micro-CT scan of the dry sandstone with a voxel resolution of 4.9 μm is derived from a publicly available data set [47,48]. For simulation purposes, a subset of 500^3^ voxels is extracted from the entire micro-CT scan (Figure 2a). The original grey-scale image is binarised based on its intensity to derive the microstructure of the sample (Figure 2b). For that purpose, the Otsu algorithm [49] is used to calculate the threshold from the histogram of the whole image stack to separate pore space and quartz grains (Figure 2c). Minor components of the Bentheim sandstone, such as kaolinite and feldspar [50] are neglected and considered to be quartz grains due to their low volumetric ratio and minor effect on the physical properties.

The entire procedure of digital image processing and analysing is done in Python using the MedPy library [51] to access the image stack. To describe the pore morphology, individual pores are extracted by means of watershed partitioning (Figure 2d). Based on this, the network including the pore and throat geometry is calculated using the PoreSpy package [52] (Figure 2e). In the following, the morphology of the pore space is described by the volume-equivalent diameter of the pores, the diameter of the throats, pores sphericity and connectivity. While sphericity is defined as surface area ratio of an equal-volume sphere to the original grain, the connectivity describes the number of throats associated with a pore. The considered digital rock sample of the Bentheim sandstone has an average grain size of 175 μm and a porosity of 23.4%, whereby the mean diameter of all 5316 pores is 92 μm (Figure 2f).

### 2.2. Permeability Determination and Pore Space Alteration Mechanisms

The permeability of the sample is determined by simulating pore-scale fluid flow directly on the binarised 3D image. This approach is known to predict rock permeabilities comparable to experimental measurements at larger sample scales [34,53]. The velocity field is calculated using the finite volume method by means of OpenFOAM [54], solving the governing equations of mass and momentum, respectively (1,2).

(1) ∇·ρv=0,

(2) $$\rho \left[\frac{\partial v}{\partial t}+v\cdot \nabla v\right]=-\nabla P+\mu \nabla^{2}v,$$

where **v** denotes the vector of the velocity field and *P* the pressure. The density and dynamic viscosity of the fluid are ρ and μ, respectively. Assuming a steady-state flow with dominating viscous forces (Reynolds number << 1) and an incompressible fluid [55], the governing equations are reduced to the simplified Stokes Equations (3) and (4):

(3) ∇·v=0,

(4) $$\mu \nabla^{2}v=\nabla P,$$

The flow velocity field is calculated by applying constant pressure boundaries at the inlet and outlet, whereas for the remaining model boundaries and internal grain wall no-slip boundary conditions are considered as in Raeini et al. [42]. Hence, the permeability (*k*) in the direction of flow can be calculated by the volume average of the velocity field ($v_{m}$) using Darcy’s law (5):

(5) $$k=\frac{v_{m}\;\mu \;l}{\Delta P},$$

where *l* is the sample length across which the pressure difference is applied. The complete permeability tensor is obtained by three simulation runs in the particular flow directions, whereby all permeability values refer to the average of the three components $k_{x}$, $k_{y}$ and $k_{z}$ in the following.

**Figure 3:**
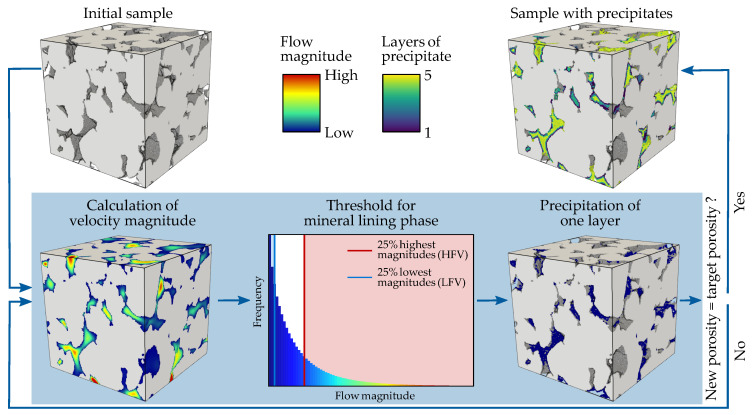
Schematic workflow of the iterative velocity-dependent precipitation process: A threshold is calculated based on the initially simulated magnitude of flow velocities. The grain-lining voxels comprising the 25% highest (HFV) or 25% lowest (LFV) flow velocity magnitudes are converted from void to mineral. Then, the flow field is calculated for the new microstructure, and the process is repeated until a pre-defined porosity is achieved or the digital sample becomes impermeable.

The extent to which changes in porosity affect permeability strongly depends on the location of mineral precipitation, since it changes the pore morphology and thus the flow parameters. It is important to note that the aim of the present study is to examine the general bandwidth of hydraulic and mechanical property changes due to dissimilar pore-scale precipitation patterns, and not to simulate a specific chemical process of mineral nucleation and growth. Hence, the alteration of the pore space is implemented by a voxel-based algorithm converting void to mineral based on the local flow velocity field without the explicit consideration of fluid-rock interactions. Godinho et al. [19] observed preferential precipitation of barite minerals at grain surfaces that were exposed to stronger fluid flow, since these regions exhibited an assured reactant replenishment. A similar behaviour was determined by Fazeli et al. [18] using reactive transport models, whereas other studies described a uniform mineral growth around the grains [56,57]. Therefore, in this study, the precipitation of minerals is correlated with the fluid flow velocity. The magnitude of the flow velocity is calculated for all three flow directions, since this reflects the full pore morphology and further considers the 3D connectivity of the pore space. Three scenarios depending on the flow field are examined: preferential mineral growth at grain surfaces exposed to (1) HFV magnitudes, (2) LFV magnitudes and (3) uniform precipitation independent of the fluid flow. The simulated nucleation and growth of minerals is an iterative process, which is illustrated in Figure 3. At first, the magnitude of the flow field for the entire pore space is calculated. Then, a threshold is set considering only the grain-lining phase, whereby voxels with the (1) 25% highest and (2) 25% lowest flow velocity magnitudes were converted from void to mineral. As the pore space is altered, fluid flow simulations are performed on the resulting microstructure. The entire procedure is iteratively repeated until a pre-defined porosity of 5% is achieved or the sample becomes impermeable.

### 2.3. Calculation of Elastic Rock Properties

**Figure 4:**
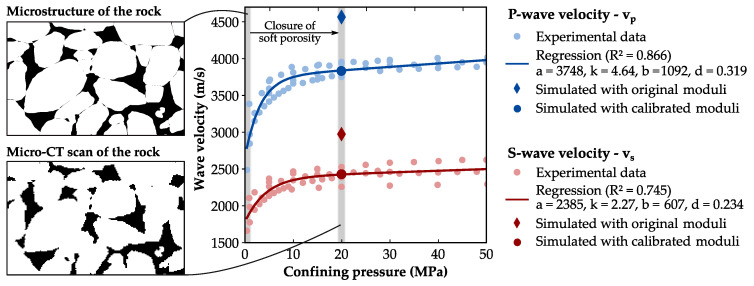
Pressure-dependent laboratory measurements [68,69,70,71,72] of dynamic elastic properties (dots) and the non-linear regression using the empirical relation of Eberhart-Phillips et al. [73] (lines) indicate an exponential increase with rising confining pressures for the Bentheim sandstone until the soft porosity features are closed. The initial mineral moduli of the grain phase are calibrated according to the workflow of Saenger et al. [71], assuming that the effect of the soft porosity features becomes negligible at 20 MPa.

The elastic properties of the digital rock sample are determined by a parallel version of the static finite element method of Garboczi and Day [43]. This well-established approach [58,59,60] computes the elastic moduli of composite materials directly from the 3D image by treating each voxel as a trilinear element. For that purpose, a small uniform strain is imposed on all voxels of the microstructure, assuming periodic boundary conditions. The algorithm solves a variational formulation of the linear elastic equations by minimizing the elastic energy [61]. The effective bulk and shear moduli are determined by the average stresses considering the six stress components of the symmetric tensor. In general, the numerically-predicted elastic moduli of granular rocks overestimate the laboratory measurements. A major reason for this mismatch is the inability of the micro-CT scan to entirely resolve the complex microstructure, particularly small pores, micro-cracks and grain-to-grain contacts [62,63,64]. These soft porosity features lead to a pressure dependency of elastic properties for granular rocks [60,65]. Thereby, seismic wave velocities exponentially increase with rising confining pressures, and the rock becomes stiffer due to the closure of the soft porosity features (Figure 4). The initial value of the mineral phase is another reason for the overestimation of elastic properties by numerical models. Since quartz grains can also contain micro cracks and small inclusions, the grain moduli may be significantly lower than those listed in Mavko et al. [66], as Mahabadi et al. [67] demonstrated for quartz grains by micro-indentation testing.

Since the soft porosity features are not resolved in the micro-CT, Saenger et al. [71] suggest to calibrate the numerical model by laboratory measurements based on the assumption that the effect of soft porosity becomes negligible at confining pressures above 20 MPa. They further implemented a pressure-sensitive grain-contact phase and linearly reduced the respective moduli to replicate the wave velocity–pressure dependency of a sandstone. In the present study, the quartz moduli are reduced according to the workflow of Saenger et al. [71] to avoid a significant overestimation of the initial elastic rock properties. For that purpose, five published datasets of laboratory measurements on the Bentheim sandstone [68,69,70,71,72] are used to perform a non-linear regression of the wave velocities to the empirical relation of Eberhart-Phillips et al. [73] (Equation (6); see Figure 4).

(6) $$v=a+kP-be^{-dP},$$

where *v* represents the P- or S-wave velocity, *P* is the confining pressure and *a*, *k*, *b*, *d* are fitting coefficients. The mineral moduli of the quartz phase are calibrated using this formulation (Table 1). Changes in elastic rock properties due to the growth of secondary minerals are investigated considering calcite as precipitate, as calcite precipitation represents a prevalent geological process and is further relevant within the context of CO_2_ storage [74] or engineering of geothermal systems [75]. The precipitated calcite phase consist of various individual crystals due to mineral nucleation and growth. Hence, the internal structure of the precipitated phase is not comparable to a compact, single crystal. For this reason, it is assumed that the precipitated calcite phase exhibits lower elastic moduli than determined for the pure mineral [66], so that the mechanical properties of amorphous calcite [76] are used in the present study.

## 3. Results

### 3.1. Morphology of the Pore Space

**Figure 5:**
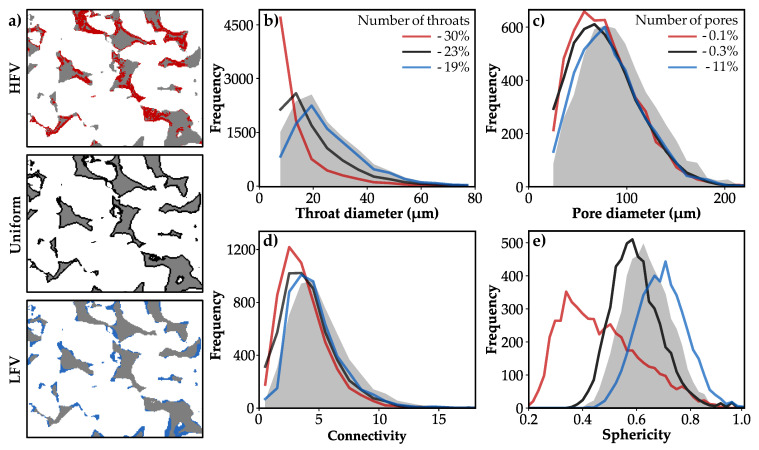
(**a**) Spatial alteration patterns at a porosity of 15.3% for preferential precipitation at high flow velocity magnitudes (HFV, red), low flow velocity magnitudes (LFV, blue) and uniform alterations, independent of fluid flow (black). Pore morphology for the three precipitation scenarios (coloured lines) and the initial pore structure (grey-filled) are described by (**b**) throat and (**c**) pore diameter, (**d**) connectivity, as well as (**e**) sphericity.

The applied precipitation mechanisms lead to distinctive spatial alteration patterns, depending on the local fluid flow velocity (Figure 5a). Changes in the morphology of the pores can be quantified by the parameters introduced in Section 2.1: throat and pore diameter, connectivity as well as sphericity. In the following, variations in rock morphology are compared for the initial state (ϕ = 23.4%) and the altered microstructure at a porosity of 15.3%, since the sample is rendered impermeable at lower porosities for the HFV scenario.

In case of precipitation in regions of HFV, 30% of the initial pore throats are completely closed and the sizes of all throats decrease substantially (Figure 5b), whereby the median pore throat diameter strongly reduces from 22 μm to 9 μm (Table 2). This indicates a predominant clogging of pore throats, whereas the absolute number of pores is not significantly reduced by precipitation (−0.1%). The pore diameter distribution becomes more right-skewed and the number of smaller pores considerably increases (Figure 5c). This demonstrates a comparably lower reduction in the diameters of smaller pores than of pores larger than circa 90 μm, even though all pore sizes are affected by precipitation. As a consequence of the major changes in pore throat diameters, the connectivity strongly reduces from 4.6 to 3.2 (Table 2), which constitutes the greatest reduction of all scenarios. In particular, the number of better connected pores decreases, whereas the number of pores associated with less than four throats increases noticeably (Figure 5d). Considerable changes in the sphericity of pores depict alterations of their shape and surface roughness (Figure 5e). The reduced mean sphericity of 0.47 for the HFV case illustrates an increase in the pore surface area due to the irregular shape and rougher structure of the precipitation pattern (Figure 5a).

The uniform precipitation mechanism continuously alters the pore structure, which demonstrates the shift of the pore and throat size distributions to lower diameters without a change in their shapes (Figure 5b,c). The median pore throat diameter reduces to 16 μm (Table 2) and 23% of the initial throats are closed. Particularly the number of smaller throats increases, since a progressive uniform precipitation leads to non-uniform changes of the pore space due to the successive clogging of throats. The median pore diameter decreases to 76 μm, and only 0.3% of all pores completely fill with precipitate. Compared to the HFV case, the pore connectivity shows a lower, but still notable reduction to 3.6 throats per pore. The mean sphericity of pores slightly decreases from 0.64 to 0.6 (Table 2), since the distribution curve shifts to lower values without changing its shape (Figure 5e).

Precipitation in regions of low flow velocities exhibits contrasting effects on pore morphology compared to the HFV case. The average throat diameter only slightly changes, since mineral growth is constrained to smaller pore throats (<30 μm) at low flow velocities. A smaller amount of 19% of the initial pore throats is clogged completely. Moreover, this scenario exhibits the least reduction in pore size, where the average diameter reduces from 92 μm to 82 μm, only. Mineral growth affects all pore sizes, but occurs more frequently in smaller pores (<90 μm) compared to a uniform alteration, whereby 11% of the initial pores are completely clogged. Precipitation in the pore space does not substantially affect the connectivity of the entire network, since the mean value is only slightly reduced to 4.2 (Table 2). Particularly the number of less-connected pores decreases predominantly. Regarding the sphericity of the pores, the surface area is smoothed and the average sphericity increases to 0.7 in the LFV scenario, since the sphericity distribution shifts to higher values and exhibits a wider spread (Figure 5e).

The most remarkable differences between the three considered scenarios occur in the number and size distributions of the pore throats, with the highest reduction in the HFV case and minor changes considering precipitation in LFV regions. Changes in pore throats directly impact the connectivity of the pore network, whereby an equivalent trend is observed. The diameters of larger pores are comparably reduced for all three precipitation patterns, whereas a contrasting effect occurs for pores smaller than 90 μm. The sphericity significantly differs between all three precipitation mechanisms. It decreases in the HFV case due to the irregular shape and rougher pore structure, while it increases in the LFV scenario in which the surface area is smoothed.

### 3.2. Flow Field and Permeability

**Figure 6:**
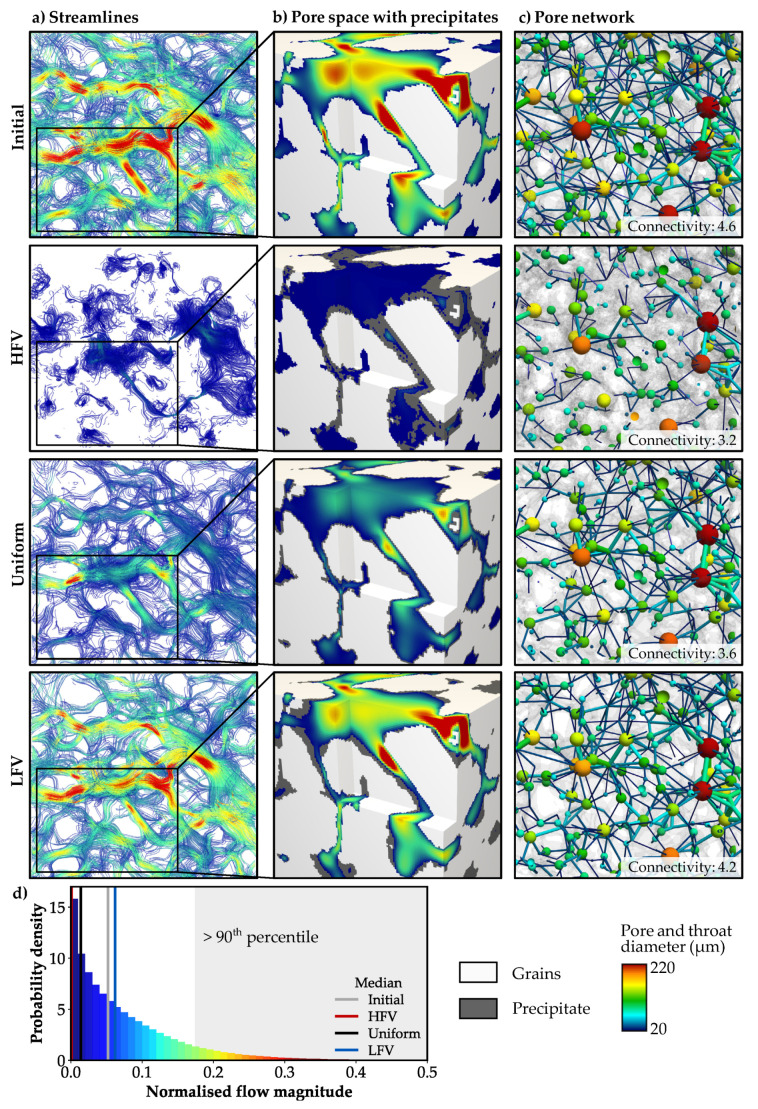
Flow regime of the initial microstructure compared to the three spatial alteration patterns. (**a**) Streamlines for flow in x-direction coloured by the velocity magnitude. (**b**) Changes in velocity magnitude relate to the location of precipitation (grey) and the resulting alteration in the (**c**) connectivity of the pore network. (**d**) Histogram of the normalised flow velocity magnitude for the initial microstructure including the changes of the median flow magnitude for the three spatial alteration patterns.

The initial flow regime within the pore space of the Bentheim sandstone is highly variable with fluid velocities varying over several orders of magnitudes. The histogram in Figure 6d illustrates the exponential decrease in probability density with increasing flow magnitudes. Hence, high flow velocities occur only at a few areas of the pore space, since the 10% of the voxels comprising the highest velocity magnitudes (>90th percentile) cover more than 80% of the entire velocity range. In general, fluid flow velocities are higher in the centre of pores; however, this does not only depend on the morphology of particular pores, but also on their connectivity. Hence, there are some regions with preferential flow paths as illustrated by the streamlines in Figure 6a.

The simulated pore alteration mechanisms with their particular morphological changes strongly impact the fluid flow paths and velocities. In case of preferential precipitation in HFV regions, a majority of the initial flow paths are cut-off (Figure 6a) due to predominant clogging of pore throats (Figure 6b). As a consequence, the pore space is considerably less connected (Figure 6c), and the median of the normalised flow velocity is substantially reduced by more than seven orders in magnitude for a decrease in absolute porosity to 15.3% (Table 3). Considering a uniform mineral growth around the grains, the connectivity of the pore network is less affected and the main fluid flow paths persist (Figure 6a). Nevertheless, the median fluid flow velocity decreases by 72%. In the LFV case, the flow regime as well as the pore network remain almost unaffected. The median of the normalised fluid flow velocities increases by 22%, compared to the initial state (Figure 6d), since areas with low flow velocities are preferentially filled with minerals.

**Figure 7:**
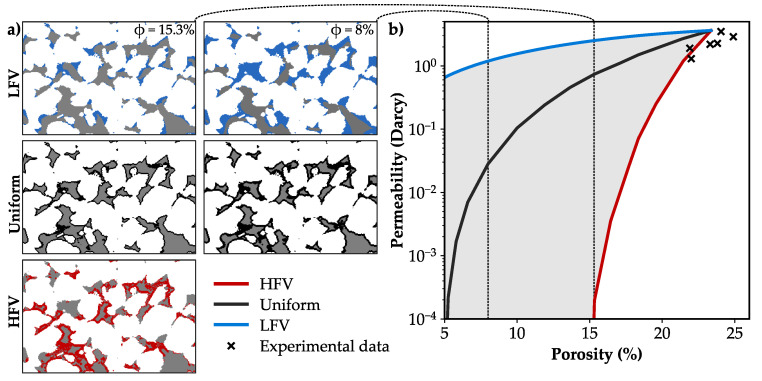
(**a**) Spatial alteration patterns at porosities of 15.3% and 8% for the preferential precipitation at high flow velocity magnitudes (HFV, red), low flow velocity magnitudes (LFV, blue) and uniform alterations, independent of fluid flow (black). (**b**) Permeability calculations of the unaffected microstructure are in good agreement with published laboratory data [22,46,77]. Permeability reduction depends on the spatial precipitation pattern and varies by up to four orders in magnitude (grey filled).

The calculated permeability of the unaffected microstructure is in good agreement with the published data for the Bentheim sandstone (Figure 7b). In case of preferential precipitation in regions of HFV, the permeability notably decreases to about 0.2 Millidarcy. Hence, the sandstone becomes nearly impermeable due to the clogging of pore throats, which agrees well with the low effective porosity of 3.5% (Table 3). Uniform pore alteration reduces the permeability by 80% to 0.74 Darcy and results in values comparable to the HFV case at a substantially lower porosity of 5.3% (Figure 7b). In case of mineral growth in regions of LFV, the permeability slightly decreases by 41% to 2.5 Darcy for a porosity of 15.3%. Even at a low porosity of 5%, the sample is still relatively permeable with 0.6 Darcy. The development of the porosity-permeability relation strongly depends on the spatial pore alteration patterns (Figure 7a), whereby the reduced permeability shows variations of up to four orders in magnitude for a porosity of 15.3%.

### 3.3. Elastic Rock Properties

**Figure 8:**
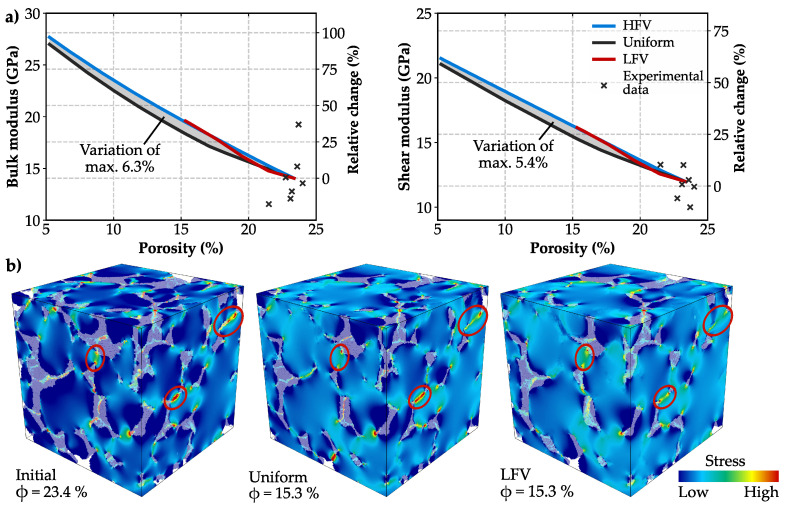
(**a**) Absolute and percentual increases in bulk and shear moduli due to preferential precipitation at high flow velocity magnitudes (HFV, red), low flow velocity magnitudes (LFV, blue) and uniform alterations, independent of the fluid flow (black). Calculated elastic properties are in good agreement with published laboratory data [68,69,70,71,72,78,79]. (**b**) Stress distribution within the initial microstructure compared to the uniform and LFV alteration patterns. Stress peaks are observed at the grain contacts (red circle).

The calculated elastic rock properties of the initial microstructure based on the calibrated bulk and shear moduli for the grain phase are in good agreement with published laboratory data for the Bentheim sandstone [68,69,70,71,72,78,79] as demonstrated in Figure 8a. The growth of minerals within the pore space generally leads to a stiffening of the rock, what depends on the elastic properties of the precipitated phase, which is calcite in the present study. While porosity is obviously the major parameter determining bulk and shear moduli, the three considered spatial precipitation patterns have only a minor effect on the increase in the elastic rock properties. For a reduction in porosity to 15.3%, both scenarios simulating a flow velocity-dependent mineral growth show an increase in bulk and shear moduli by 38.6% and 34.5%, respectively (Figure 8a). Further precipitation and the resulting porosity reduction to 5% leads almost to a doubling of the initial bulk modulus and an increase in shear modulus by 80%. For the considered range of precipitation, mineral growth in regions of LFV leads to 6.3% higher effective elastic rock properties in maximum compared to a uniform coating around the grains. In case of precipitation in areas of HFV, the calculated elastic behaviour is initially comparable to the uniform pattern, but aligns to the behaviour of the LFV case with decreasing porosities (Figure 8a).

Variations in stress concentrations between the spatial precipitation patterns are associated with the grain contacts. In general, stress concentrates at the grain-to-grain interfaces, as it is clearly visible for the initial microstructure (Figure 8b). Despite their completely different impact on hydraulics, both fluid flow velocity-dependent precipitation patterns enhance the grain contacts. As shown in Figure 8b, the stress distributes more evenly within the granular microstructure, so that the sandstone behaves slightly stiffer in the LFV scenario compared to a uniform alteration pattern.

## 4. Discussion

The three considered scenarios assume a preferential precipitation depending on the magnitude of fluid flow velocity and can be associated with general contrasting chemical processes. A predominant mineral growth near pore throats, where fluid flow velocities are relatively high (HFV), represents a transport-controlled process. Thereby, advection dominates and the reaction is limited by the availability of reactants transported to the fluid-mineral interface [74], which in turn depends on the local flow regime. Consequently, minerals precipitate preferentially in regions with higher flow velocity due to higher solute flux. By contrast, uniform mineral growth around the grains represents a surface reaction-controlled process [74], whereby the chemical reaction rate limits precipitation and the pore space is altered homogeneously. Both spatial precipitation patterns have been observed in several laboratory [19,21,56] and numerical studies [18,80,81], investigating mostly calcite and barite precipitation in porous media. Preferential precipitation in regions of LFV can be explained by an undisturbed growth of minerals, whereby fluid shear stresses limit precipitation. However, such an effect has been observed within the context of biomass accumulation, only [82,83].

### 4.1. Range of the Porosity-Permeability Relation

The location of mineral growth is the major controlling factor for the reduction in permeability. Hence, the porosity–permeability relationship represents the dominant process for pore space alteration. Permeability evolution due to mineral precipitation or dissolution can be described by various analytical and empirical relations. Hommels et al. [84] reviewed existing approaches and demonstrated that many of these lead to predictions that are comparable to the power law relation with an appropriate exponent (Equation (7)). Thereby, the ratio of the current (*k*) and initial permeability ($k_{0}$) is related to the ratio of the current (ϕ) and initial porosity ($\phi_{0}$) with an exponential fitting factor (η).

(7) $$\frac{k}{k_{0}}={\left(\frac{\phi }{\phi_{0}}\right)}^{\eta }$$

Another model to describe the permeability evolution is the Kozeny–Carman relation [28,85], which was initially developed to estimate the permeability for a grain pack without considering pore alteration processes (Equation (8)). Nevertheless, it is widely applied to describe changes in permeability due to the precipitation and dissolution of minerals [86,87,88], and for that reason used for comparison against the results of the present study.

(8) $$k=\frac{S^{2}d^{2}}{180}\frac{\phi^{3}}{\left(1-\phi^{2}\right)},$$

where *S* is the sphericity and *d* the average grain diameter of 175 μm for the considered sample of the Bentheim sandstone.

**Figure 9:**
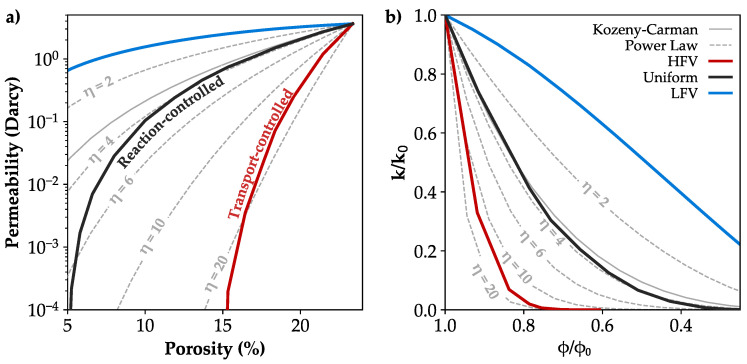
(**a**) Simulated permeability decrease due to preferential precipitation at high flow velocity magnitudes (HFV, red), low flow velocity magnitudes (LFV, blue) and independent of the fluid flow (black) compared to porosity-permeability estimates by the Kozeny–Carman (grey line) and the power law relations with different exponents η (dashed lines). (**b**) Porosity-permeability relations normalised to their initial values.

A transport-controlled process represented by the HFV case is characterised by clogging of pore throats, and hence a substantial decrease in pore space connectivity. Particularly larger pores are affected by precipitation, and the reduction in pore diameter is limited to a certain size, which is indicated by the considerable increase in smaller pores. This can be explained by a successive disconnection of pores from the high velocity flow field, and thereby the availability of reactants. The resulting strong permeability reduction can be roughly described by the power law with an exponent of 20 (Figure 9a). A surface reaction-controlled precipitation represented by the uniform pattern exhibits a continuous alteration of the pore structure, whereby all pores are equally affected, but progressive precipitation induces non-uniform changes in the pore network due to a successive clogging of pore throats. It corresponds approximately to the power law with an exponent of four. Published values for η within the context of mineral precipitation comprise values between 11 [89], 8 [90] and 6 [81], while Nogues et al. [81] found variable exponents depending on the fluid composition, flow rate and porosity for a synthetic carbonate. These findings lie in the range of the simulated contrasting cases of a uniform mineral growth and preferential precipitation at high flow velocities, which demonstrates the applicability of the simulated reaction- and transport-controlled precipitation process applied in the present study. Niu and Zhang [80] even found a higher value of η = 30, considering a transport-limited mineral growth and concluded that a constant exponent cannot describe this process sufficiently well due to the strong permeability decrease. The Kozeny–Carman relation initially exhibits a good agreement with a uniform mineral growth around the grains. However, at lower porosities the pore space is altered in an increasingly non-uniform manner due to the successive clogging of pore throats resulting in an overestimation of the permeability by the Kozeny–Carman model (Figure 9a).

Precipitation in regions of low fluid velocities occurs preferentially in smaller throats and pores with a minor impact on the flow regime. This concerns low velocity regions of larger pores as well as particularly smaller pores, which are poorly connected. Moreover, the increase in sphericity represents a reduction in pore roughness and a smoothing of the mineral surfaces. Hence, the initial fluid flow regime is only slightly affected, and the resulting porosity-permeability relation exhibits a power law exponent considerably below two (Figure 9b), which is generally associated with diagenetically altered rocks, only [90,91]. Therefore, it is concluded that a predominant mineral growth in regions of LFV is not a representative natural process of precipitation within the pore space.

### 4.2. Impact on Elastic Rock Properties

**Figure 10:**
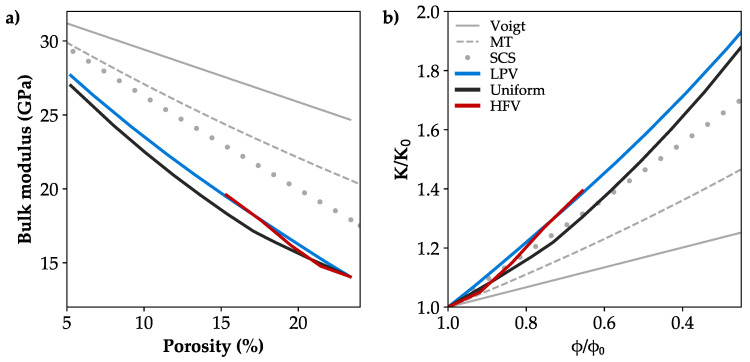
(**a**) Simulated increase in bulk moduli due to preferential precipitation at high flow velocity magnitudes (HFV, red), low flow velocity magnitudes (LFV, blue) and a uniform grain coating (black) compared to analytical solutions (grey) as the Voigt average, Mori–Tanaka approach (MT), and the Self-Consistent Scheme (SCS). (**b**) Stiffening trend by normalisation of bulk moduli (K) and porosity (ϕ).

The examined velocity-dependent spatial alteration patterns have only a limited impact on the increase in elastic rock properties, while porosity is clearly the controlling parameter for the effective bulk and shear moduli. This is because precipitation alters the pore space, but does not significantly affect the already consolidated granular structure of the sandstone at the same time. The prediction of elastic rock properties by means of a 3D microstructural representation of the rock is substantially improved in this study, compared to state-of-the-art analytical methods (Figure 10a), where bulk moduli are overestimated by up to 75% for the Voigt average [92], 46% for the Mori–Tanaka approach [33] and 27% for the Self-Consistent Scheme [32]. These three homogenisation methods were discussed in more detail in Wetzel et al. [93]. While Mori–Tanaka’s approach and the Voigt average underestimate the increase in elastic properties, the stiffening trend of approximately 0.63 GPa and 0.47 GPa per percent porosity decrease for bulk and shear moduli, respectively, can be roughly approximated by the Self-Consistent Scheme (Figure 10b).

Since the location of mineral growth does not considerably affect the stiffening behaviour of the consolidated sandstone, as indicated by the variations of 6.3% in maximum, a detailed characterisation of the spatial alteration pattern is not required. The increase in bulk and shear moduli can be described by an easy-to-implement uniform grain coating or analytically by the Self-Consistent Scheme, presuming an adequate estimation of the initial elastic rock properties. Nevertheless, a stronger effect of precipitation patterns may arise for unconsolidated material or considering diagenetic processes, where mainly grain contacts are affected by stiffening [31,94,95]. Moreover, even though precipitation of minerals generally causes a stiffening of the rock, it can also lead to a contrasting behaviour. Zhang et al. [96] determined a significant decrease in elastic moduli by up to 21% due to salt precipitation in a tight sandstone, where secondary cracks developed and weakened the rock matrix. Moreover, Noiriel et al. [97] examined sodium chloride precipitation in a fractured sandstone and observed the evolution of fracture networks developed by the nucleation of microcracks.

This study considers calcite as precipitating mineral, as it is relevant within the context of subsurface utilisation [74,75]. Nevertheless, the main findings of this study are generally applicable regarding the changes in physical rock properties due to precipitation, since the choice of calcite does neither influence the spatial precipitation patterns nor the reduction in permeability. Solely the calculated elastic properties are affected by the moduli of calcite. Thus, higher bulk and shear moduli of the precipitating phase would result in a higher stiffening. However, the initial moduli only slightly influence the computed effective rock moduli compared to the changes in porosity, as demonstrated by Wetzel et al. [93], who examined the effect of stiff as well as soft moduli for a calcite cement. Hence, both the general stiffening trend, as well as the variations between the spatial precipitation scenarios are transferable to other commonly precipitating elastic minerals.

### 4.3. Added Value of Using a Velocity-Dependent Precipitation Algorithm

The results of this study demonstrate that the location of precipitation within the pore space is of paramount importance for the permeability reduction, whereas the impact on the elastic rock properties is limited. Hence, to predict the effect of precipitation especially on the hydraulic rock behaviour, an adequate characterisation of the spatial pore alteration pattern is crucial. Of course, the process of mineral nucleation and growth is complex and depends on more factors than solely the fluid flow velocity: the location of mineral precipitation is controlled by the chemical reaction regime, which depends on fluid chemistry [16,17], transport properties [18,19], mineralogy [20,21], temperature [22,23] and pore morphology [24,25]. Hence, in contrast to reactive transport simulations, the presented approach cannot examine the temporal aspect of precipitation, e.g., the clogging of pores near the inlet, as can be observed in pore-scale laboratory experiments [16,98]. Moreover, the pore roughness is not specifically considered in the present study. Precipitated mineral layers often exhibit a certain roughness depending the location, shape and size of the precipitation [21], whereby an increase in pore roughness reduces the permeability.

Despite these limitations, the presented approach offers a simplified approximation of the spatial alteration pattern depending on the geochemical reaction regime without the requirement of implementing complex reactive transport simulations. The magnitude of the fluid flow velocity considers the specific pore morphology as well as the overall connectivity, and thereby represents regions with reactant replenishment. Hence, the range of permeability decrease between surface reaction-limited (uniform case) and transport-controlled precipitation (HFV case) can be determined for a representative elementary volume of a granular rock. Moreover, the calculated porosity-permeability relations, as well as the increase in mechanical properties are transferable to reservoir-scale models and can describe the physical rock properties as a consequence of mineral precipitation, e.g., in the context of reservoir engineering to determine the amount of precipitate leading to a considerable reduction in productivity or injectivity. For a specific application, it would be reasonable to determine whether the particular geochemical process is mainly reaction- or transport-controlled. Thereby, the range of uncertainty in the porosity-permeability relation can be substantially reduced.

## 5. Summary and Conclusions

The presented approach numerically examines the impact of mineral precipitation on hydraulic and mechanical properties for a granular reservoir rock. A micro-CT scan of the Bentheim sandstone is used as a highly resolved digital rock sample. The permeability is determined from the flow field by solving the steady-state Stokes equation, whereas effective rock stiffness is calculated by a static finite element method. To investigate the impact of the location of precipitation on pore morphology, permeability and elastic moduli, the initial microstructure is altered by preferential precipitation at high flow velocity magnitudes, low flow velocity magnitudes and a uniform mineral growth around the grains.

The results of this study demonstrate that the location of precipitation strongly affects the permeability with variations by up to four orders in magnitude, whereas the impact on elastic rock properties is limited with differences of 6.3% in maximum. The number and size of pore throats, and hence the connectivity of the pore network are mainly responsible for the distinctive permeability reduction resulting from the investigated pore space alterations. Preferential precipitation in regions of high velocity magnitudes can be associated with a transport-controlled reaction regime. It is characterised by a predominant clogging of pore throats, resulting in a drastic reduction in overall connectivity of the pore network. Hence, the permeability strongly decreases, whereby the porosity-permeability relation can be roughly described by the power law with an exponent of 20. A uniform mineral growth around the grains is associated with reaction-controlled precipitation, whereby the size of all pores is equally reduced. The reduction in permeability is less pronounced and can be approximated by the power law with an exponent of four or the Kozeny–Carman relation. Preferential mineral growth in regions of low flow velocities affects predominantly smaller throats and pores with a minor impact on the flow regime. Hence, the reduction in permeability is considerably below the power law with an exponent of two, and it is concluded that this scenario does not represent a natural process of precipitation within the pore space.

The stiffening trend in bulk and shear moduli amounts circa 0.63 GPa and 0.47 GPa per percent porosity decrease, respectively. Porosity is clearly the controlling parameter for the increase in effective bulk and shear moduli. Moreover, small variations between the spatial alteration patterns are associated with the grain contacts, whereby both flow velocity-dependent precipitation patterns enhance the grain contacts, resulting in a slightly stiffer behaviour than the uniformly altered sample. Hence, a detailed consideration of the spatial alteration pattern is not required and the increase in elastic moduli can be approximated by an easily-implementable uniform grain coating, whereas an adequate characterisation of pore alteration patterns is crucial to quantify the reduction in hydraulic rock parameters.

The presented approach offers a simplified estimation of spatial pore space alteration for surface reaction-limited and transport-controlled geochemical reaction regimes without the requirement of implementing complex reactive transport simulations. Digital rock physics in view of a virtual laboratory can be used to quantify changes in effective rock properties for different rock microstructures or chemical reaction regimes. Hence, the calculated porosity-permeability relations as well as changes in mechanical properties can be applied in reservoir-scale numerical simulation models to improve their predictive capabilities, which is of paramount importance for an improvement of processes understanding and a sustainable utilisation of the geological subsurface.

## Figures and Tables

**Table 1:** Bulk and shear moduli for all minerals used to compute effective elastic rock properties.

Mineral	Bulk Modulus (GPa)	Shear Modulus (GPa)	Reference
Quartz	37.0	44.0	[66]
Quartz (calibrated)	32.2	28.3	Present study
Calcite (amorphous)	35.5	14.0	[76]
Pore space	0	0	

**Table 2:** Morphological parameters of the initial pore space compared to the microstructures altered by the three spatial precipitation patterns (HFV, uniform and LFV).

Scenario	Porosity (%)	Throat Diameter (μm)	Pore Diameter (μm)	Connectivity	Sphericity
Initial	23.4	22	92	4.6	0.64
HFV	15.3	9	78	3.2	0.47
Uniform	15.3	16	76	3.6	0.60
LFV	15.3	23	82	4.2	0.70

**Table 3:** Hydraulic properties of the initial pore space compared to the microstructures altered by the three spatial precipitation patterns (HFV, uniform and LFV).

Scenario	Absolute Porosity (%)	Effective Porosity (%)	Median of Normalised Flow Magnitude	Permeability (Darcy)
Initial	23.4	23.3	5.2 × 10${}^{-2}$	3.63
HFV	15.3	3.5	3.7 × 10${}^{-9}$	2 × 10${}^{-4}$
Uniform	15.3	15.2	1.4 × 10${}^{-2}$	0.74
LFV	15.3	15.3	6.4 × 10${}^{-2}$	2.52
